# Supplementary material for: A pathogenic variant in RAB32 causes autosomal dominant Parkinson’s disease and activates LRRK2 kinase
Source: medRxiv. 2024 Jan 18:2024.01.17.24300927. Preprint. [Version 1] doi: 10.1101/2024.01.17.24300927 (PMC10827257; doi:10.1101/2024.01.17.24300927)
Supplement: Supplement 1 [file media-1.pdf]

Table of Contents

SUPPLEMENTARY METHODS 2

Pathologic diagnoses 2

Whole exome sequencing (WES), variant selection and genotyping 2

Haplotype analysis 2

Structural modelling 2

RAB32 mRNA and protein expression across tissues 3

Cell culture and constructs 3

Quantitative immunoblot analysis 4

Antibodies used for quantitative immunoblotting analysis 4

Immunocytochemistry and co-localization 4

Image processing and Analysis 5

SUPPLEMENTARY TABLES 6

SUPPLEMENTARY FIGURES 13

SUPPLEMENTARY REFERENCES 20

SUPPLEMENTARY METHODS

Pathologic diagnoses

The brain was removed within 24 hours of death, with one hemisphere frozen at 80 °C, the other fixed in formalin, and regions embedded in paraffin for dissection and histologic studies to provide a pathologic diagnosis of parkinsonism, as previously described[37].

Whole exome sequencing (WES), variant selection and genotyping

Exonic regions were enriched using the Ion AmpliSeq exome kit (57·7Mb) and sequenced on the Ion Proton (Life Technologies, Carlsbad, CA, USA) with a minimum average coverage of 70 reads per base and an average read length of 150 bases. Reads were mapped to the NCBI Build 37·1 (hg19) reference genome using the Ion Torrent Suite 5·0. Sequences with a mapping Phred quality score under 20, fewer than 10 reads or over 95% strand bias were excluded from further analysis. Variants were annotated with ANNOVAR[1] and Combined Annotation Dependent Depletion (CADD) C-scores[2] to help rank functional, deleterious and disease causal variants.

Variants were selected for subsequent analysis if they were: (1) good quality calls, greater than 10 reads deep with balanced forward and reverse reads; (2) predicted to be SNVs or insertions/deletions (indels) in an exonic or splicing region; (3) had a minor allele frequency (MAF) of ≤ 0·01 from the genome Aggregation Database (gnomAD; http://gnomad.broadinstitute.org/), and; (4) were absent in our in-house control exomes.

Subsequent genotyping was performed by Sanger sequencing of specific exons, as previously described[3], or using TaqMan probes (Life Technologies), following the manufacturer's instructions.

Haplotype analysis

Single nucleotide polymorphisms (SNP) were genotyped in the Tunisian families (TUN I and TUN II) using Affymetrix 500K NspI and StyI ships, and genotypes were extracted from .cel intensity files using three algorithms, BBRML, JAPL and CHIAMO, and only included when there was consensus. One patient was genotyped on the HumanCyto-12_300K chip (FRA1). Otherwise, Illumina Multi-Ethnic Genome Arrays (MEGA) were used in all other familial probands and the French-Canadian family (CAN I) (**Figure 1**); GenomeStudio® was used to provide genotypes for Illumina data. DNA from all available family members and samples identified with the putatively pathogenic RAB variant were genotyped, as specified above. Alternatively, genotypes for the same SNPs were retrieved from whole genome data using bcftools. Phase was established within pedigrees when possible.

Structural modelling

AlphaFold modelling[4] was used to predict the interaction between LRRK2 and RAB32. A local installation of ColabFold[5] using MMseqs2 sequence alignment and AlphaFold2-multimer-v3 model, with additional AMBER structure relaxation was employed to model interactions between the LRRK2 fragments (1-1000 or 350-550, Uniprot: Q5S007) and RAB32 (Ser71 WT and Arg71 mutant, Uniprot: Q13637). Resulting structures were visualized and analyzed using

PyMOL 2·5·5. BIOVIA Discovery Studio Visualizer 2021 was used to determine possible intermolecular interactions (Non-bond Interaction Monitor) and to predict potential rotamers.

RAB32 mRNA and protein expression across tissues

For *RAB32* mRNA expression we used RNA-seq data for 17,510 human samples originating from 54 different human tissues (GTEx, v8) that were downloaded using the R package recount (v 1·4·6)Click or tap here to enter text.. Cell lines, sex-specific tissues, and tissues with 10 samples or below were removed. Samples with large chromosomal deletions and duplications or large copy number variation previously associated with disease were filtered out. For RAB32 protein expression we downloaded normal tissue data from Human Protein Atlas https://www.proteinatlas.org/, accessed on 02/08/2023). In addition, we performed immunohistochemical staining in C57BL/6 mice to assess Rab32 expression in TH-expressing cells of the SNpc. Immunohistochemistry was performed, as previously describedClick or tap here to enter text., using primary rabbit anti-mouse RAB32 (ABC520, Millipore; 1:500) and chicken anti-Tyrosine Hydroxylase (ab76442, Abcam; 1:1000). Secondary antibodies used were Alexa Fluor® Goat anti-rabbit, chicken or mouse IgG (H+L) 488 and 568 secondary (1:1000).

Cell culture and constructs

HEK293FT cells were maintained in Dulbecco's Modified Eagle Medium (DMEM, Gibco) supplemented with 10% Heat Inactivated Fetal Bovine Serum (ThermoHEK293 cells (RRID: CVCL_0045) were purchased from the American Type Culture Collection and maintained in DMEM containing 10% (v/v) FBS, 2 mM l-glutamine, penicillin (100 U/ml), and streptomycin (100 µg/ml). All cells were grown at 37°C temperature with 5% CO_2 in a humidified atmosphere and regularly tested for mycoplasma contamination. The following plasmids were used for cell transfection and kinase activity assays: HA-empty vector (DU49303); HA-RAB32 Ser71 wild-type (DU52622); HA-RAB32 Arg71 (DU77801); HA-RAB32 Ala71 (DU77802); HA-RAB29 wild-type (DU50222); Flag-LRRK2 wild-type (DU62804). These were generated by the MRC PPU Reagents and Services at the University of Dundee (https://mrcppureagents.dundee.ac.uk). Each construct was confirmed by sequencing at the MRC Sequencing and Services (https://www.dnaseq.co.uk). All plasmids are available to request via the MRC PPU Reagents and Services website (https://mrcppureagents.dundee.ac.uk). A detailed description of cell transfection (dx.doi.org/10.17504/protocols.io.bw4bpgsn) and cell lysis methods (dx.doi.org/10.17504/protocols.io.b5jhq4j) have previously been described. Briefly, HEK293 cells were seeded into 6-well plates and transiently transfected at 60-70% confluence using Polyethylenimine (PEI) transfection reagent (Polysciences, Inc., #24765). For each well, 1·6 µg of N-ter Flag-tagged LRRK2 (wild-type or mutant), 0·4 µg of N-ter HA-tagged Rab32 or Rab29 (wild-type, mutant or control) (or HA-empty vector) and 6 µg of PEI were diluted in 0·5 mL of Opti-MEM™ Reduced serum medium (Gibco™) and incubated for 15-20 minutes at room temperature before being added to the cells. Cells were lysed 24 hrs post-transfection in ice-cold lysis buffer containing 50 mM Tris-HCl pH 7·4, 1 mM EGTA, 10 mM 2-glycerophosphate, 50 mM sodium fluoride, 5 mM sodium pyrophosphate, 270 mM sucrose, supplemented with 1 µg/ml microcystin-LR, 1 mM sodium orthovanadate, complete EDTA-free protease inhibitor cocktail (Roche), and 1% (v/v) Triton X-100. Lysates were clarified by centrifugation at 17,000 g at 4 °C for 10 min and supernatants were quantified by Bradford assay.

Quantitative immunoblot analysis

A detailed description of the quantitative immunoblotting protocol has previously been described (dx.doi.org/10.17504/potocols.io.bsgrnby6). Briefly, cell lysates were mixed with a quarter of a volume of 4 x SDS-PAGE loading buffer (Invitrogen™ NuPAGE™ LDS Sample Buffer, cat# NP0007) and heated at 70 °C for 5 min. Samples were loaded onto NuPAGE 4–12% Bis–Tris Midi Gels (Thermo Fisher Scientific, Cat# WG1402BOX or Cat# WG1403BOX) and electrophoresed at 130 V for 2 hrs in NuPAGE MOPS SDS running buffer (Thermo Fisher Scientific, Cat# NP0001-02). Proteins were then electrophoretically transferred onto a nitrocellulose membrane (GE Healthcare, Amersham Protran Supported 0·45 μm NC) at 90 V for 100 min on ice in transfer buffer (48 mM Tris base and 39 mM glycine supplemented with 20% (v/v) methanol). The membranes were blocked with 5% (w/v) skim milk powder dissolved in TBS-T (50 mM Tris base, 150 mM sodium chloride (NaCl), 0·1% (v/v) Tween 20) at room temperature for 1 hr before overnight incubation at 4 °C in primary antibodies. Membranes were washed three times for 15 min each with TBS-T before being incubated with secondary antibodies for 1 hr at room temperature. Thereafter, membranes were washed with TBS-T three times with a 15-minute incubation for each wash, and protein bands were acquired via near-infrared fluorescent detection using the LI-COR Odyssey CLx Western Blot imaging system, and intensities of bands were quantified using Image Studio Lite (version 5·2·5, RRID:SCR_013715).

Antibodies used for quantitative immunoblotting analysis

The antibodies against Rab10 Thr73 [MJF-R21] (ab230261) and LRRK2 Ser1292 [MJFR-19-7-8] (ab203181) and the anti-HA tag antibody (ab18181) were purchased from Abcam. The rabbit monoclonal antibody against LRRK2 Ser935 (UDD2) was purified by MRC PPU Reagents and Services at the University of Dundee (https://mrcppureagents.dundee.ac.uk). The mouse monoclonal antibody against total LRRK2 (C-terminus) was purchased from NeuroMab (clone N241A/34, #75-253). The mouse monoclonal against total Rab10 was purchased from Nanotools (#0680–100/Rab10-605B11). All primary antibodies were diluted in 5% (w/v) bovine serum albumin (BSA) in TBS-T and used at a final concentration of 1 μg/ml.

Goat anti-mouse IRDye 680LT (#926-68020) and goat anti-rabbit IRDye 800CW (#926-32211) secondary antibodies were from LI-COR and were diluted 1:20,000 (v/v) in 5% (w/v) milk in TBS-T.

Immunocytochemistry and co-localization

For experiments where GFP-RAB32 localization was examined, HeLa cells seeded on glass coverslips in 24-well plates were transfected with 1ug of cDNA using Lipofectamine 2000 (Invitrogen). The following day, coverslips were fixed, processed and imaged as previously described(16). For co-transfection of GFP-RAB32, HA-PINK1 and mCherry-Parkin, HEK293 cells were seeded on glass coverslips (Poly-D-Lysine coated) at a density of ~50,000 cells/well in 24-well plates in DMEM media without antibiotics supplemented with 10% FBS (heat inactivated). After 24 hours, cells were co-transfected using lipofectamine 3000 with plasmid constructs as follows: (a). GFP-Rab32 WT (0·5 μg) and HA-PINK1 WT (1·0 μg) and mCherry-Parkin (1·0 μg); (b). GFP-Rab32 WT (0·5 μg) and HA-PINK1 kinase dead (KD) (1·0 μg) and mCherry-Parkin (1·0 μg); (c) GFP-Rab32 S71R (0·5 μg) and HA-PINK1 WT (1·0 μg) and mCherry-Parkin (1·0 μg); and (d) GFP-Rab32 S71R (0·5 μg) and HA-PINK1 KD (1·0 μg) and mCherry-Parkin (1·0 μg). 24 hours post-transfection, cells were washed in 1x phosphate

buffered solution (PBS) (pH 7·4) and fixed in 10% Formalin (buffered saline, pH 7·4) for 20 minutes at room temperature and permeabilized in 0·1% triton X-100 in 1x PBS for 15 minutes at room temperature with gentle shaking followed by blocking in 10% normal donkey serum in 1x PBS for 1 hour. Cells were incubated with primary mouse mAb anti-HA tag (Thermo-scientific Pierce: PI26183) antibody (1:8000) overnight at 4°C. Cells were washed 3 times for 5 minutes each wash in 1x PBS. Secondary donkey anti-mouse Alexa Fluor 647 antibody (1:1000) was added to the cells and incubated for 2 hours at room temperature with gentle shaking. Primary and secondary antibodies were diluted in 0·02% triton X-100, 1% normal donkey serum in 1x PBS. Cells were washed 3 times for 5 minutes each wash in 1x PBS and mounted using Prolong Diamond anti-fade media (Life Technologies) and allowed to dry overnight before processing for confocal imaging. To assess co-localization between Rab32 and PINK1, a Leica TCS SP5 laser scanning confocal microscope (Leica Microsystem) was used. High resolution images were acquired using 63x oil-immersion (scan averaged, four times; 1024 × 1024 pixel resolution). Initial settings for pinhole, digital gain and noise reduction were optimized and all acquisition parameters were kept the same throughout the imaging. Co-localization was analyzed by Pearson's correlation co-efficient (ImageJ plug-in JACoP). 12-15 cells with comparable co-transfection efficiency were selected for co-localization analysis. Statistical analysis was performed using two-way ANOVA (Graph pad Prism 8·1·1) and presented as mean ± SEM (p ≥0·0001).

Image processing and Analysis

Transfected cells were imaged under oil-immersion at 60x magnification on an Olympus FV-1000 confocal laser scanning microscope (9 x 0·33µm step size). Images were stacked using ImageJ software (NIH, USA), and masks were created to encompass the entirety of a cell, excluding the nucleus as labelled by DAPI. Masks and non-manipulated images were processed using a custom pipeline with Cell Profiler software (v·2·1·1) to identify number and density of GFP-RAB32 (-Ser71Arg)-positive structures between 1-3µm in diameter.

SUPPLEMENTARY TABLES

Supplementary tables 1.

A) Clinical Characteristics of 130 probands with Parkinson's disease

**Clinical characteristics**	
No. of families/Probands	130
Female sex, No. (%)	47 (36.2)
Male sex, No. (%)	83 (63.8)
Age at onset (y)	
Mean ± SD	54.2 ± 14.1
Range	13-83
Age (y)	
Mean ± SD	72.7 ± 11.7
Range	38-96
Race/Ethnicity	
Caucasian	109
East Asian	2
Hispanic	1
North African	18

B) Case control series

	Tunisia		Caucasian	
	PD	Controls	PD	Controls
Number of Patients	400	344	2204	-
Mean Age at Onset (AAO)	55.2±14.7	-	60.2±11.8	-
Mean Age	-	66.3±11.1	-	-
Age Range	13-87	39-100	10-80	-
Gender Ratio (M:F)	1:1	1.1:1	1.7:1	-

C) PD database resources

	AMP-PD		GP2		German clinical diagnostic database		100,000 Genomes Project
	PD	Controls	Familial/Young Onset PD	Controls	Parkinsonism	non-PD	Familial/Young Onset PD
Number of Patients	3105	3670	1849	349	311	23389	778
Mean Age at Onset (AAO)	-	-	-	-	-	-	-
Mean Age	-	-	-	-	-	-	-
Age Range	-	-	-	-	-	-	-

Gender Ratio (M:F)	-	-	-	-	-	-	-

Supplementary table 2. RAB GTPase gene sequencing depth from whole-exome analysis.

Gene	Position (hg19)	RefSeq	Average sequencing depth ± SD
RAB42	chr1:28918711-28921088	NM_001193532	101.5 ± 102.3
RAB3B	chr1:52373627-52456436	NM_002867	195 ± 109.5
RAB13	chr1:153954092-153958853	NM_002870	117.4 ± 120.1
RAB25	chr1:156030965-156040295	NM_020387	128.4 ± 94
RAB29 (RAB7L1)	chr1:205737113-205744610	NM_003929	128.4 ± 123.7
RAB4A	chr1:229406808-229441640	NM_004578	86.4 ± 97.5
RAB18	chr10:27793102-27831166	NM_021252	108.2 ± 93.2
RAB1B	chr11:66036055-66044963	NM_030981	186.2 ± 146
RAB6A	chr11:73386682-73472201	NM_002869	76.4 ± 51.6
RAB30	chr11:82684174-82782965	NM_001286059	165.5 ± 101.9
RAB38	chr11:87846414-87908635	NM_022337	107.2 ± 76.7
RAB39A	chr11:107799276-107834208	NM_017516	127.9 ± 88.3
RAB5B	chr12:56367696-56390467	NM_002868	134.3 ± 115.2
RAB21	chr12:72148657-72181150	NM_014999	77.1 ± 57.7
RAB35	chr12:120532898-120554643	NM_006861	136.4 ± 74.5
RAB20	chr13:111175412-111214071	NM_017817	183.6 ± 143.4
RAB2B	chr14:21927178-21945132	NM_032846	123.9 ± 107.7
RAB15	chr14:65412531-65438875	NM_198686	142.4 ± 86.5
RAB27A	chr15:55495163-55582013	NM_004580	91.1 ± 82.2
RAB8B	chr15:63481727-63559973	NM_016530	73.7 ± 66.8
RAB11A	chr15:66161796-66184329	NM_004663	103.9 ± 83.4
RAB40C	chr16:639356-679273	NM_001172663	134.6 ± 97.2
RAB26	chr16:2198650-2204141	NM_014353	107.5 ± 94.2
RAB34	chr17:27041298-27045286	NM_031934	155.2 ± 129.8
RAB5C	chr17:40276993-40307062	NM_004583	152.4 ± 73.6
RAB37	chr17:72667255-72743474	NM_001163989	135.6 ± 109.2
RAB40B	chr17:80614942-80656598	NM_006822	105.4 ± 77.5
RAB12	chr18:8609442-8639380	NM_001025300	68.6 ± 41.1
RAB31	chr18:9708227-9862553	NM_006868	153.6 ± 94
RAB27B	chr18:52495707-52562747	NM_004163	91.6 ± 45.9
RAB11B	chr19:8455204-8469317	NM_004218	108.2 ± 103.9
RAB3D	chr19:11432721-11450344	NM_004283	115 ± 97
RAB8A	chr19:16222489-16244445	NM_005370	125.6 ± 85.5
RAB3A	chr19:18307610-18314874	NM_002866	153.7 ± 100.1
RAB4B	chr19:41284123-41302849	NM_016154	161 ± 91.2
RAB10	chr2:26256728-26360323	NM_016131	95.5 ± 59.9

RAB1A	chr2:65313987-65357435	NM_004161	87.6 ± 57.3
RAB6C	chr2:130737234-130740311	NM_032144	169.6 ± 81.3
RAB17	chr2:238482964-238499769	NM_022449	171.8 ± 71.1
RAB22A	chr20:56884770-56942563	NM_020673	128.9 ± 111.9
RAB36	chr22:23487512-23506531	NM_004914	154.6 ± 95.6
RAB5A	chr3:19988571-20026667	NM_004162	98.4 ± 91.2
RAB7A	chr3:128444978-128533641	NM_004637	153.8 ± 113.6
RAB43	chr3:128806411-128840993	NM_198490	163.3 ± 134.3
RAB6B	chr3:133543079-133614691	NM_016577	154.8 ± 110.3
RAB28	chr4:13369346-13485989	NM_001159601	75.9 ± 66.9
RAB33B	chr4:140374960-140397069	NM_031296	126.4 ± 89.5
RAB3C	chr5:57878938-58147406	NM_138453	130.2 ± 63.1
RAB24	chr5:176728190-176730745	NM_001031677	174.5 ± 98.6
RAB44	chr6:36665627-36700960	NM_001257357	NA
RAB23	chr6:57051790-57087112	NM_016277	93 ± 84.1
RAB32	chr6:146864827-146876086	NM_006834	146.1 ± 234
RAB19	chr7:140103842-140126050	NM_001008749	183.2 ± 96.2
RAB2A	chr8:61429468-61536203	NM_002865	117.5 ± 148.6
RAB14	chr9:123940414-123964365	NM_016322	105.8 ± 63
RAB9A	chrX:13707239-13727944	NM_004251	87.3 ± 44.4
RAB41	chrX:69502021-69504852	NM_001032726	98.2 ± 90.8
RAB40A	chrX:102754680-102774417	NM_080879	120.4 ± 68.8
RAB9B	chrX:103077254-103087212	NM_016370	80.9 ± 61.8
RAB33A	chrX:129305772-129318844	NM_004794	77.9 ± 60.6
RAB39B	chrX:154487518-154493874	NM_171998	92.8 ± 68.6

Supp. table 3. 15 rare non-synonymous variants in the Rab GTPase genes in 130 probands with PD or primary parkinsonism.

Gene	Chr:Position (hg19)	Ref/Alt	RefSeq	NT change	AA change	rsID	ExAC frequency	gnomAD frequency	CADD phred score	No. of carriers	Zygosity
RAB3B	1:52385700	T/C	NM_002867	c.559A>G	p.Met187Val	rs34017695	2.00e-04	2.53e-04	27.8	1/130	Het
RAB17	2:238483760	G/T	NM_022449	c.541C>A	p.Leu181Met	rs112742374	2.00e-04	2.47e-04	35	1/130	Het
RAB6B	3:133553479	G/A	NM_016577	c.502C>T	p.Arg168Ter	rs147187493	8.25e-06	8.12e-06	44	1/130	Het
RAB28	4:13373484	A/C	NM_001159601	c.581T>G	p.Phe194Cys	NA	NA	NA	25	1/130	Het
RAB24	5:176729484	T/C	NM_001031677	c.347A>G	p.Y116Cys	NA	NA	NA	23.4	1/130	Het
RAB32	6:146865220	C/G	NM_006834	c.213C>G	p.Ser71Arg	rs200251693	3.43e-05	2.45e-05	19.2	3/130	Het
RAB38	11:87908390	G/A	NM_022337	c.163C>T	p.Pro55Ser	rs769358206	9.19e-06	4.08e-06	35	1/130	Het
RAB27A	15:55497812	G/A	NM_004580	c.559C>T	p.Arg187Trp	rs144946000	1.00e-04	1.55e-04	24.5	1/130	Het
RAB27A	15:55516087	C/G	NM_004580	c.467G>C	p.Gly156Ala	rs200031368	1.65e-05	2.89e-05	41	1/130	Het
RAB8B	15:63548776	A/T	NM_016530	c.397A>T	p.Lys133Ter	NA	NA	NA	19.03	1/130	Het
RAB26	16:2201727	G/A	NM_014353	c.380G>A	p.Arg127H	rs149935646	4.00e-04	4.08e-04	34	1/130	Het
RAB37	17:72733173	G/A	NM_001163989	c.25G>A	p.Gly9Arg	rs762798603	1.00e-04	1.23e-04	16.52	1/130	Het
RAB40B	17:80616378	C/T	NM_006822	c.554G>A	p.Arg185Gln	rs199886901	3.00e-04	2.78e-04	13.67	1/130	Het
RAB12	18:8636320	A/G	NM_001025300	c.586A>G	p.Ile196Val	rs143888944	5.00e-04	3.03e-04	19.12	1/130	Het
RAB36	22:23503707	G/A	NM_004914	c.958G>A	p.Glu320Lys	rs9624038	6.00e-04	7.10e-04	33	1/130	Het

Supplementary table 4. RAB32 locus genotyping and the RAB32 Arg71 consensus haplotype

Variant rsID	Chr:Position (hg19)	Ref	Alt	*MAF	TUN1	TUN2	CAN1	TUN3	CAN2	POL1	ITL1	FRA1	UK1	UK2	AMP1	AMP2	AMP3	AMP4	CAN3
rs11155451	6:146422524	A	G	**0.38**	**G**	**G**	**G**	A/**G**	A	A	A/**G**		**G**	A/**G**	**G**	A/**G**	A	A/**G**	**G**
rs362949	6:146652354	T	C	**0.09**	**T**	**T**	**T**		**T**	**T**	**T**				**T**	**T**	**T**/C	**T**	**T**
rs56566	6:146669045	T	C	**0.27**	**C**	**C**	**C**	T/**C**	**C**	**C**	T/**C**	T/**C**	T/**C**		T/**C**	T/**C**	T	T/**C**	T/**C**
rs362845	6:146680907	G	T	**0.12**	**G**	**G**	**G**	**G**	**G**	**G**/T	**G**	**G**			**G**/T	**G**	**G**/T	**G**	**G**
rs362848	6:146682485	A	G	**0.10**	**A**	**A**	**A**	**A**	**A**	**A**	**A**				**A**	**A**	**A**	**A**/G	**A**
rs2300619	6:146685324	T	C	**0.01**	**T**	**T**	**T**	**T**	**T**	**T**	**T**	**T**			**T**	**T**	**T**	**T**	**T**
rs362852	6:146712761	T	C	**0.43**	**T**	**T**	**T**	**T**	**T**	**T**/C	**T**	**T**	**T**/C	**T**/C	**T**/C	**T**/C	**T**/C	**T**	**T**
rs1358902**	6:146828922	A	G	**0.23**	**G**	**G**	**G**	**G**	**G**	A/**G**	**G**		**G**	**G**	**G**	**G**	A/**G**	**G**	**G**
rs9497566**	6:146849283	C	T	**0.20**	**T**	**T**	**T**	**T**	**T**	**T**/C	**T**	**T**	**T**/C	**T**/C	**T**	**T**	**T**	**T**	**T**
rs719787**	6:146849677	T	C	**0.29**	**C**	**C**	**C**	T/**C**		T/**C**		**C**	**C**	T/**C**	**C**	**C**	T/**C**	**C**	**C**
RAB32 p.S71R	6:146865220	C	G		**G**	**G**	**G**	**G**	**G**	**G**	**G**	**G**	**G**	**G**	**G**	**G**	**G**	**G**	**G**
rs2275606	6:146918950	G	A	**0.08**	**A**	**A**	**A**	G/**A**	G/**A**	G/**A**	G/**A**		G/**A**	G/**A**	G/**A**	G/**A**	**A**	G/**A**	G/**A**
rs9497607	6:147013320	G	T	**0.12**	**G**	**G**	**G**	**G**	**G**/T	**G**	**G**/T				**G**	**G**/T	**G**/T	**G**	**G**
rs4896886	6:147053632	C	T	**0.18**	**T**	**T**	**T**	**T**	**T**/C	**T**	**T**/C	**T**	**T**/C	**T**/C	**T**/C	**T**/C	C	**T**/C	**T**/C
rs6937015**	6:147187237	T	C	**0.36**	**C**	**C**	**C**	**C**	**C**	**C**/T	**C**	**T**/C	**T**/C	**C**	**C**	**C**	**T**/C	**C**	**T**/C
rs9390434	6:147189967	T	C	**0.46**	**T**	**T**	**T**	**T**	C	**T**/C	C		C/**T**	C	C	**T**/C	**T**/C	C	**T**/C

Gametic phase for haplotypes in TUN1, TUN2 and CAN1 were determined from their pedigrees (Figure 1). Otherwise allele calls from genotyping is shown. *MAF=Minor allele frequency (dbSNP ALFA). Nevertheless, the minor allele by frequency is given for the Ref allele for variant rsIDs**. The minimal consensus haplotype in gray = 0.361 Mb

Supplementary table 5. RAB32 variants in all samples

**AMP-PD**	**UK Biobank**	**GP2 variants**
Met1Arg	Ala2Thr	Leu34Val
Ala2Thr	Gly10Arg	Asn68Thr
Gly5Glu	Leu25Phe	**Ser71Arg**
Ala13Val	Gly32Arg	Tyr96Cys
Leu49Phe	Ile42Met	Phe114Ser
Ala63Asp	Lys43Met	Asp123Asn
Val66Ile	Leu49Pro	Pro155Ser
Asn68Thr	His53Arg	Glu164Gly
**Ser71Arg**	Asn68Thr	Trp170Cys
Gln85Lys	**Ser71Arg**	
Tyr96Cys	Arg87Ter	
Gly101Arg	Tyr96Cys	
Glu115Gly	Asp123Asn	
Asp123Asn	His129Leu	
Gly169Arg	Lys149Arg	
Trp170Cys	Gln160Glu	
Lys191Asn	Gly169Arg	
Arg87X	Trp170Cys	
Trp120X	Lys191Thr	
	Leu216Ser	

X=stop codon/frameshift. Variant frequencies in PD versus control participant frequencies within each database were compared, and only RAB32 Ser71Arg was found to be significantly increased in PD cases in the AMP-PD database (as reported in the main text). Data on other RAB32 variants was not available from German clinical diagnostic databases although they revealed four heterozygotes. One male patient with PD is documented in the main text, but 3 other young female heterozygotes were identified with non-neurologic disorders. Their symptoms included the: 1) immune system, with abnormal inflammatory responses; 2) proteinuria, a nephropathy consisting of focal segmental glomerulosclerosis, and; 3) Tetralogy of Fallot with pulmonary stenosis. Diagnostic testing was performed at 22, 15 and 15 years of age, respectively, but no other clinical nor genetic data was available.

SUPPLEMENTARY FIGURES

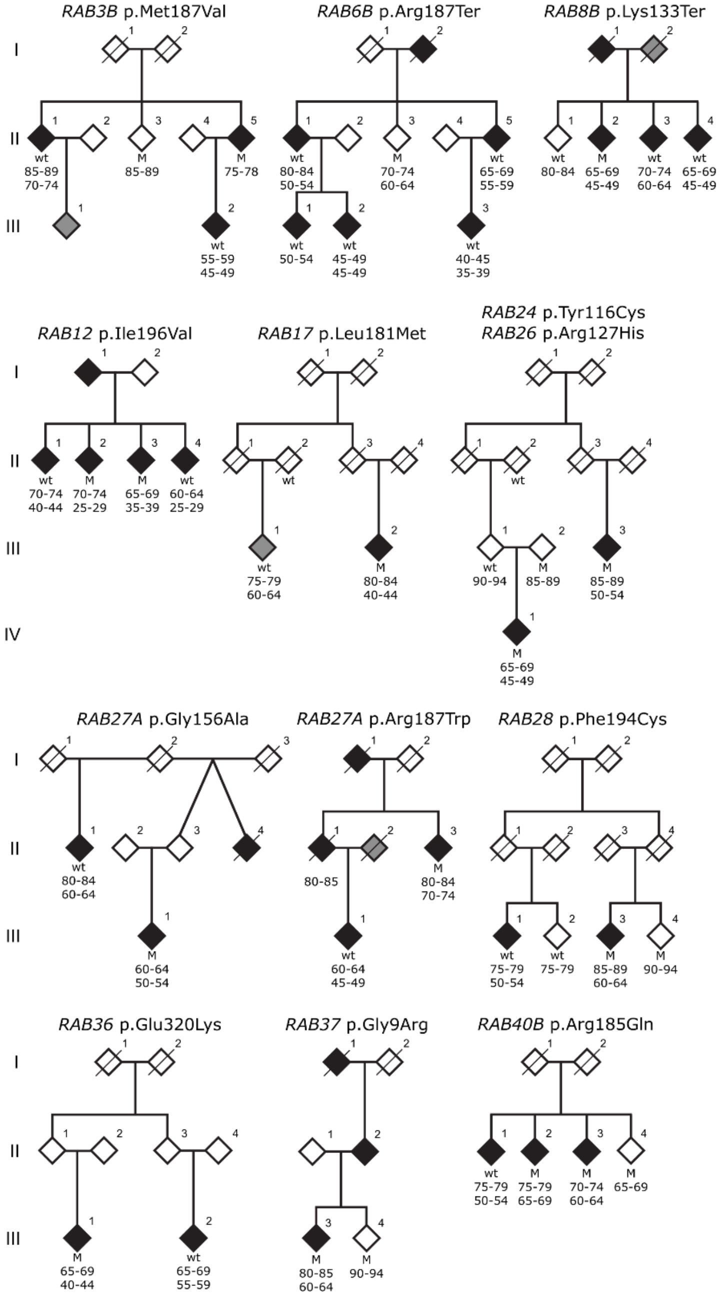

Supplementary figure 1. Pedigrees with multi-incident parkinsonism. Individual pedigrees are labeled by the variant identified in the proband. 'M' denotes a carrier of the specific variant, and wt stands for wild type. Age (top) and, when available, age at onset (bottom) is denoted under genotype. Black filled symbols denotes PD and grey filled symbols other neurological symptoms.

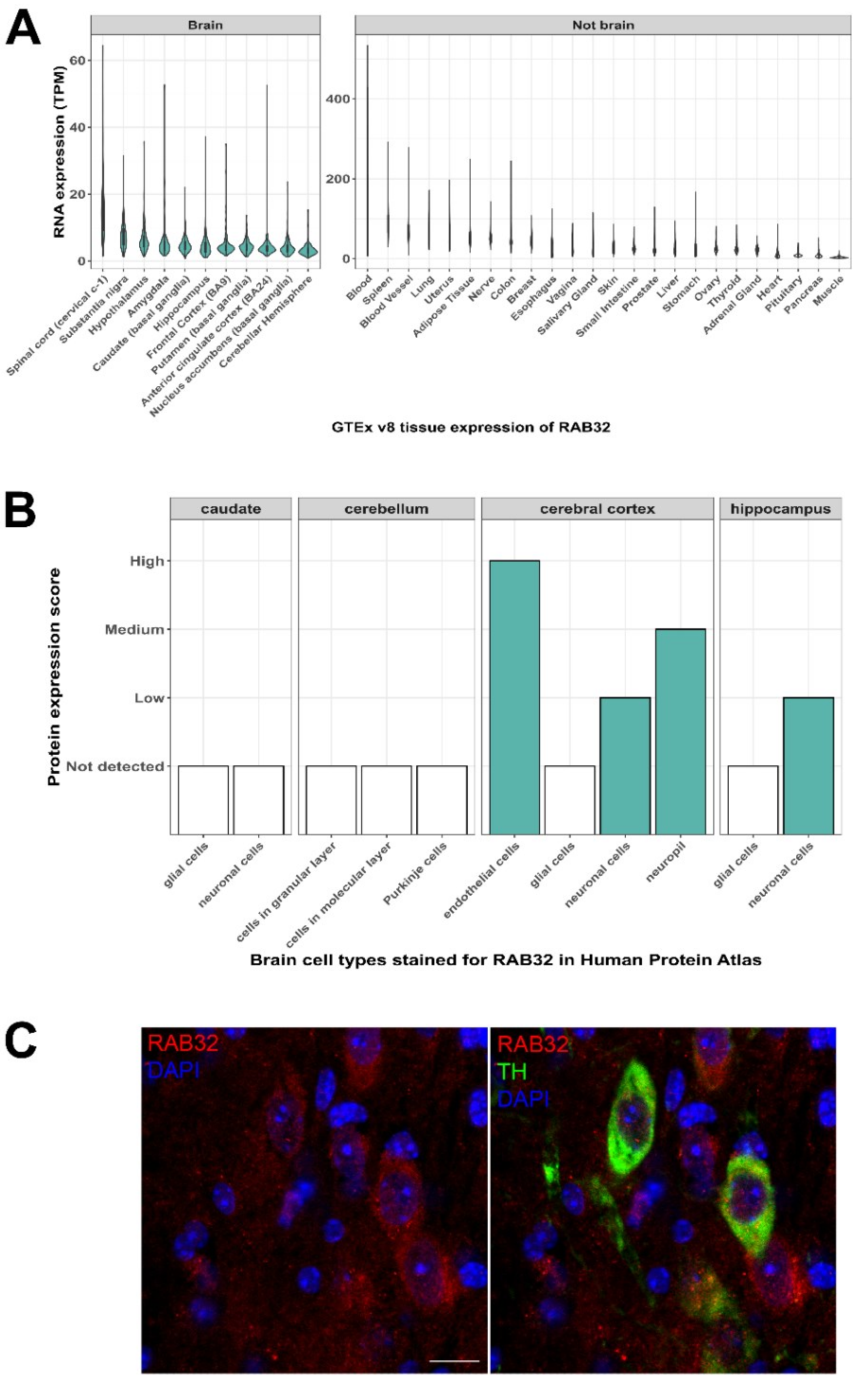

Supplementary figure 2. A) Transcripts per million (TPM) expression of RAB32 across human tissues generated by the Genotype-Tissue Expression Consortium, GTEx v8). **B)** RAB32 protein expression in brain tissues through Immunohistochemical staining. Data generated by Human Protein Atlas. **C)** Immunohistochemical staining in 3 month old male mice revealed that RAB32 is expressed in TH-expressing neurons of the *substantia nigra pars compacta*.

Homo sapiens	ATIGVDFALKVLNWDSRTLVRLQLWDIAGQE
Mus musculus	ATIGVDFALKVLNWDSRTLVRLQLWDIAGQE
Danio rerio	ATIGVDFALKVLNWDSKTLVRLQLWDIAGQE
Xenopus tropicalis	ATIGVDFALKVINWDSSTLVRLQLWDIAGQE
Rattus norvegicus	ATIGVDFALKVLNWDSRTLVRLQLWDIAGQE
Macaca mulatta	ATIGVDFALKVLNWDSRTLVRLQLWDIAGQE
Bos tauru	ATIGVDFALKVLNWDSRTLVRLQLWDIAGQE
Gallus gallus	ATIGVDFALKVINWDSKTLVRLQLWDIAGQE
Sus scrofa	ATIGVDFALKVLNWDSRTLVRLQLWDIAGQE
Microcebus murinus	ATIGVDFALKVLNWDSRTLVRLQLWDIAGQE
Felis catus	ATIGVDFALKVLNWDSRTLVRLQLWDIAGQE
Esox lucius	ATIGVDFALKVINWDSKTLVRLQLWDIAGQE
Meleagris gallopavo	ATIGVDFALKVINWDSKTLVRLQLWDIAGQE
Alligator mississipienis	ATIGVDFALKVLHWDSKTLVRLQLWDIAGQE
Fukomys damarensis	ATIGVDFALKVLSWDSRTLVRLQLWDIAGQE
Serinus canaria	ATIGVDFALKVINWDSKTLVRLQLWDIAGQE
Parus major	ATIGVDFALKVINWDSKTLVRLQLWDIAGQE
Callithrix jacchus	ATIGVDFALKVLNWDSRTLVRLQLWDIAGQE
Monodelphis domestica	ATIGVDFALKVLNWDSKTLVRLQLWDIAGQE
Anolis carolinensis	ATIGVDFALKVLPWDSRTLVRLQLWDIAGQE
Poecilia reticulate	ATIGVDFALKVINWDSKTLVRLQLWDIAGQE

Supplementary figure 3. Conservation of RAB32 Ser71. Protein homologs were aligned via ClustalO. Amino acid position for RAB32 p.Ser71Arg is highlighted in black. Protein homologs with amino acid positions differing from those of the human RAB32 sequence are indicated in gray. RefSeq accession numbers: Homo sapiens (NP_006825), Mus musculus (NP_080681), Danio rerio (NP_958489), Xenopus tropicalis (NP_001011276), Rattus norvegicus (NP_001102372), Macaca mulatta (NP_001180823), Bos taurus (NP_001192879), Gallus gallus (NP_001264601), Sus scrofa (NP_001116648), Microcebus murinus (XP_012607651), Felis catus (XP_003986676), Esox lucius (XP_010901778), Meleagris gallopavo (XP_003204151), Alligator mississippiensis (XP_006271694), Fukomys damarensis (XP_010624980), Serinus canaria (XP_009091018), Parus major (XP_015478707), Callithrix jacchus (XP_002747112), Monodelphis domestica (XP_001370750), Anolis carolinensis (XP_003215798) and Poecilia reticulata (XP_008395960).

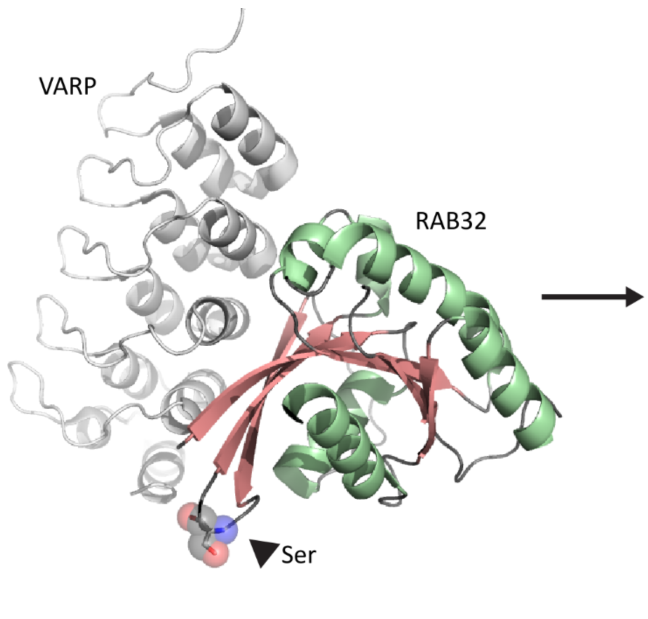

Supplementary figure 4. Crystal structure of RAB32 (PDB ID: 4cym) showing the substitution of serine to arginine at residue 71, adjacent to the VARP binding domain.

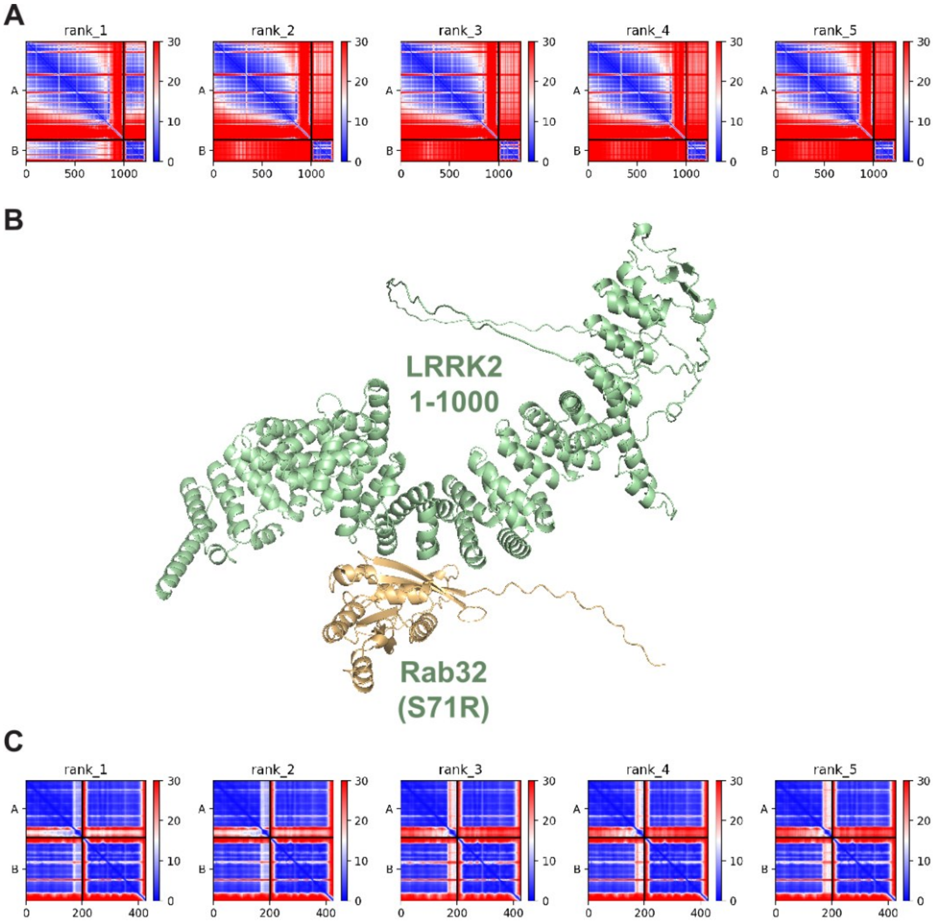

Supplementary figure 5. A) PAE plots of 5 models for the LRRK2(1-1000)/Rab32 prediction. **B)** Structure overview of the top model for the LRRK2(1-1000)/Rab32 prediction. **C)** PAE plots of 5 models for the LRRK2(350-550)/Rab32 prediction**.**

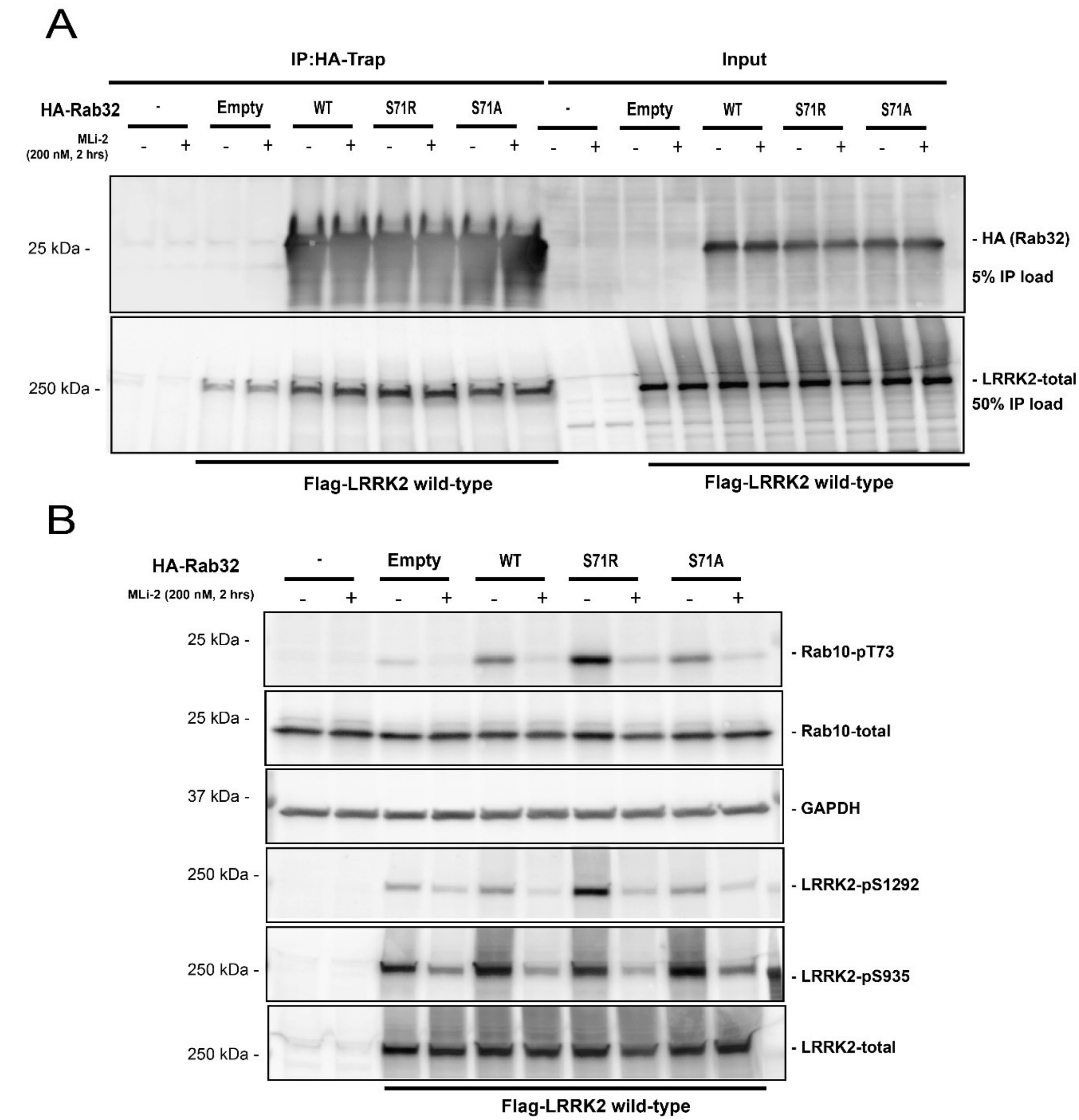

Supplementary figure 6. RAB32 co-immunoprecipitation with LRRK2. **A)** Immunoprecipitation (IP) analysis of lysates from HEK293FT cells transiently co-expressing 3xFLAG-LRRK2 and either HA empty vector, HA-RAB32 WT or mutant (p.S71R or p.S71A) cDNA. IP with HA-Trap was followed with immunoblot analysis with an anti-HA (top panel) or an anti-FLAG antibody (bottom panel) as indicated. Separate gels from the same IP sample were loaded to limit HA-signal saturation. GAPDH is used for loading control. Input denotes whole cell lysate starting material from HEK293FT cells used for immunoprecipitation analysis; +/− indicates cDNA transfections and Mli-2 treatment. B) Western blot analysis of WCL used in (A) to confirm MLi-2 target engagement was monitored by a loss of pS1292 and pS935 phosphorylation for LRRK2 and pT73 Rab10 for Rab10. MLi-2 was applied at 200nM for 120 mins prior to cell lysis.

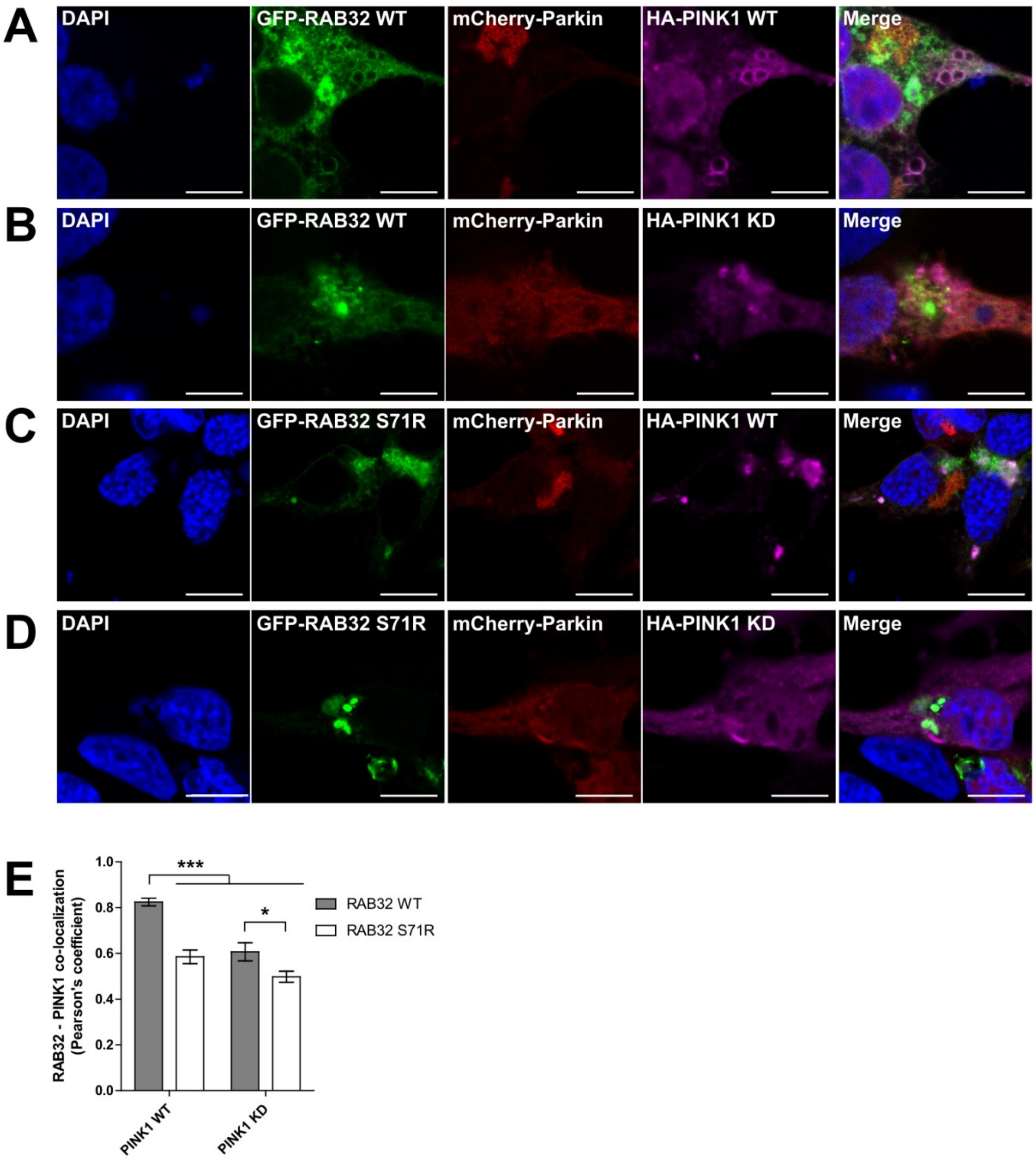

Supplementary figure 7. RAB32 co-localization with PINK1. Confocal images of HEK293 cells co-transfected with mCherry-Parkin and GFP-tagged wild-type RAB32 **(A, B)** or S71R RAB32 **(C, D)** and HA-tagged wild-type PINK1 **(A, C)** or kinase dead PINK1 **(B, D)**. Boxes indicate regions shown at higher power below. Scale bars are 10 microns. **(E)** Pearson's correlation coefficient to quantify the co-localization of GFP-RAB32 and HA-PINK1. Bars show mean +/-SEM of 12-15 fields. * indicates $P<0.05$, *** indicates $P<0.0001$ according to ANOVA followed by Tukey's multiple comparisons test.

SUPPLEMENTARY REFERENCES

1 Wang K, Li M, Hakonarson H. ANNOVAR: functional annotation of genetic variants from high-throughput sequencing data. *Nucleic Acids Res* 2010; **38**: e164–e164.

2 Kircher M, Witten DM, Jain P, O'roak BJ, Cooper GM, Shendure J. A general framework for estimating the relative pathogenicity of human genetic variants. *Nature Genetics 2014 46:3* 2014; **46**: 310–5.

3 Gustavsson EK, Trinh J, Guella I, *et al.* DNAJC13 genetic variants in parkinsonism. *Movement Disorders* 2015; **30**: 273–8.

4 Jumper J, Evans R, Pritzel A, *et al.* Highly accurate protein structure prediction with AlphaFold. *Nature* 2021; **596**: 583–9.

5 Mirdita M, Schütze K, Moriwaki Y, Heo L, Ovchinnikov S, Steinegger M. ColabFold: making protein folding accessible to all. *Nat Methods* 2022; **19**: 679–82.

6 Collado-Torres L, Nellore A, Kammers K, *et al.* Reproducible RNA-seq analysis using recount2. Nat Biotechnol. 2017. DOI:10.1038/nbt.3838.

7 Cataldi S, Follett J, Fox JD, *et al.* Altered dopamine release and monoamine transporters in Vps35 p.D620N knock-in mice. *npj Parkinson's Disease 2018 4:1* 2018; **4**: 1–11.
